# Unveiling the Role of Hormonal Imbalance in Breast Cancer Development: A Comprehensive Review

**DOI:** 10.7759/cureus.41737

**Published:** 2023-07-11

**Authors:** Shweta Satpathi, Sagar S Gaurkar, Ashwini Potdukhe, Mayur B Wanjari

**Affiliations:** 1 Internal Medicine, Jawaharlal Nehru Medical College, Datta Meghe Institute of Higher Education and Research, Wardha, IND; 2 Otolaryngology and Head and Neck Surgery, Jawaharlal Nehru Medical College, Datta Meghe Institute of Higher Education and Research, Wardha, IND; 3 Medical Surgical Nursing, Srimati Radhikabai Meghe Memorial College of Nursing, Datta Meghe Institute of Higher Education and Research, Wardha, IND; 4 Research and Development, Jawaharlal Nehru Medical College, Datta Meghe Institute of Higher Education and Research, Wardha, IND

**Keywords:** hormonal therapies, hormone receptors, progesterone, estrogen, breast cancer, hormonal imbalance

## Abstract

Breast cancer is a complex and multifactorial disease with a significant global impact. Hormonal imbalance has emerged as a crucial factor in breast cancer development, highlighting the importance of understanding the intricate interplay between hormones and breast tissue. This comprehensive review aims to unveil the role of hormonal imbalance in breast cancer by exploring the involvement of key hormones, including estrogen and progesterone, and their receptors in tumor development. The review delves into how hormonal imbalance impacts breast tissue, emphasizing the significance of hormone receptor status in guiding treatment decisions. Furthermore, the review investigates the influence of other hormones, such as insulin and growth factors, and their cross-talk with hormone pathways in breast cancer progression. The implications of hormonal imbalance assessment in breast cancer risk assessment and the importance of hormone testing in diagnosis and treatment decisions are also discussed. Moreover, the review provides an overview of the various hormonal therapies used in breast cancer treatment, their benefits, limitations, and ongoing research efforts to optimize their efficacy and overcome resistance. Future directions in hormonal therapy research, including developing novel therapies and personalized medicine approaches, are explored. This review underscores the need for a comprehensive understanding of hormonal imbalance in breast cancer to enhance prevention, diagnosis, and treatment strategies, ultimately improving outcomes for individuals affected by this disease.

## Introduction and background

Breast cancer is among the most prevalent cancers affecting women worldwide [1]. It is a complex disease with various risk factors and underlying mechanisms contributing to its development and progression. Among these factors, hormonal imbalance has significantly affected breast cancer pathogenesis. Understanding the intricate relationship between hormonal imbalance and breast cancer development is crucial for advancing our knowledge in this field and potentially improving prevention and treatment strategies [1-3].

Breast cancer is characterized by the abnormal growth of cells in the breast tissue [4]. It is the most commonly diagnosed cancer in women and the second leading cause of cancer-related deaths globally. The incidence of breast cancer varies across different populations, with certain risk factors such as age, family history, and genetic mutations predisposing individuals to a higher likelihood of developing the disease. The impact of breast cancer extends beyond physical health, affecting psychological well-being and quality of life [4,5].

Hormonal imbalance involving estrogen and progesterone has been extensively studied in breast cancer [6]. Estrogen, a hormone predominantly produced in the ovaries, plays a crucial role in the growth and development of breast tissue. Similarly, progesterone, another hormone primarily produced in the ovaries, regulates the menstrual cycle and breast development [7]. Disruptions in the delicate balance of these hormones have been implicated in the initiation and progression of breast cancer [6-9].

This comprehensive review article aims to shed light on the pivotal role of hormonal imbalance in the development of breast cancer. Through an in-depth analysis of the existing literature, this review aims to provide a comprehensive understanding of the mechanisms underlying hormonal imbalance, its impact on breast cancer initiation and progression, and its potential implications for therapeutic interventions and preventive strategies.

## Review

Methodology

To conduct this review, a comprehensive literature search was performed using several electronic databases, including PubMed, MEDLINE, and Google Scholar. The search strategy involved the use of relevant keywords related to breast cancer, hormonal imbalance, and associated concepts. Additionally, the reference lists of selected articles were examined to identify any additional relevant studies. The selection of appropriate studies was ensured through the application of specific inclusion and exclusion criteria. The inclusion criteria encompassed studies published in peer-reviewed journals that directly addressed the role of hormonal imbalance in breast cancer development. Various study designs, such as experimental studies, clinical trials, observational studies, and systematic reviews, were considered eligible for inclusion. Conversely, case reports and editorials were excluded from the review. Preference was given to English-language articles published within the last 10 years to incorporate recent research. However, seminal studies and landmark papers beyond this time frame were also included if they provided significant insights into the topic. The selection process involved an initial screening of titles and abstracts to identify potentially relevant articles. Subsequently, full-text articles were assessed based on the predefined inclusion and exclusion criteria. Any discrepancies or uncertainties encountered during the selection process were resolved through thorough discussion and consensus among the authors. By employing this rigorous methodology with explicit inclusion and exclusion criteria, this review aimed to ensure the inclusion of high-quality studies that contribute to a comprehensive understanding of the role of hormonal imbalance in breast cancer development. To facilitate clarity and better understanding, the revised version of this section will include schematics representing the inclusion/exclusion criteria and the number of articles included based on abstracts or full-text analysis.

Breast cancer: overview and risk factors

A Brief Overview of Breast Cancer, Its Types, and Stages

Breast cancer is a heterogeneous disease characterized by the uncontrolled growth of abnormal cells in the breast tissue [10-12]. Breast cancer has ranked number one among Indian females, with an age-adjusted rate of 25.8 per 100,000 women and a mortality of 12.7 per 100,000 women [1]. It can occur in both women and men, although it is much more common in women. Breast cancer can manifest in various forms, including ductal carcinoma in situ (DCIS), invasive ductal carcinoma (IDC), invasive lobular carcinoma (ILC), and less common subtypes such as inflammatory breast cancer and triple-negative breast cancer [1,4,5].

DCIS refers to abnormal cells within the milk ducts, while IDC and ILC indicate the invasion of cancer cells into the surrounding breast tissue. The stage of breast cancer is determined based on the size of the tumor, lymph node involvement, and metastasis to other organs. Staging helps in determining appropriate treatment approaches and predicting prognosis [9].

Genetic Factors, Family History, and Environmental Factors

Genetic factors play a significant role in breast cancer development. Specific gene mutations, such as mutations in the BRCA1 and BRCA2 genes, significantly increase the risk of developing breast cancer. These mutations are inherited and can be passed down from generation to generation. Individuals carrying BRCA1 or BRCA2 mutations have a higher lifetime risk of developing breast cancer and an increased risk of ovarian cancer [10,11].

In addition to BRCA1 and BRCA2, other genetic mutations, such as TP53, PTEN, and PALB2, are associated with breast cancer susceptibility [12]. Family history also plays a significant role in breast cancer risk. Research has shown that individuals with a first-degree relative, such as a mother or sister, who has been diagnosed with breast cancer, are at a higher risk of developing the disease. This information is supported by numerous studies and scientific sources, emphasizing the importance of considering familial factors in assessing an individual’s susceptibility to breast cancer. Genetic testing and counseling are essential for individuals with a strong family history of breast cancer [12,13].

Hormone Replacement Therapy

Long-term use of hormone replacement therapy (HRT), particularly combined estrogen-progestin therapy, has been associated with an increased risk of breast cancer. Women considering HRT should carefully weigh the potential benefits against the risks and consult with their healthcare provider [14,15].

Reproductive Factors

Several reproductive factors have been identified as potential risk factors for breast cancer. These factors include early onset of menstruation (before the age of 12), late onset of menopause (after the age of 55), late age at first childbirth, and nulliparity (never having given birth). Studies have shown that these factors are associated with an increased risk of breast cancer [16,17].

Alcohol Consumption

Regular and excessive alcohol consumption has been consistently linked to an increased risk of breast cancer. The risk increases with higher levels of alcohol intake. It is advisable to limit alcohol consumption or abstain from it altogether to reduce the risk of breast cancer [18,19].

Obesity and Physical Inactivity

Obesity, especially postmenopausal obesity, is a known risk factor for breast cancer. Adipose tissue produces estrogen, and higher estrogen levels can promote breast cancer development. Additionally, a lack of regular physical activity is associated with an increased risk. Maintaining a healthy weight through a balanced diet and regular physical exercise can help reduce the risk [20,21].

Environmental Exposures

Prolonged exposure to certain environmental factors has been suggested to increase the risk of breast cancer. Ionizing radiation, such as from medical imaging tests or radiation therapy, is a known risk factor. Additionally, exposure to certain chemicals and pollutants, such as those in specific workplaces or environmental contaminants, may increase risk. Minimizing exposure to such factors is essential for reducing the risk of breast cancer [22,23].

Hormonal imbalance and breast cancer

Hormonal imbalance, particularly estrogen and progesterone, has been extensively implicated in the development and progression of breast cancer [23]. Estrogen is a hormone primarily produced by the ovaries and plays a crucial role in the growth and development of breast tissue. It promotes cell proliferation and regulates gene expression in cell cycle progression and apoptosis [24]. Progesterone, an essential hormone primarily produced in the ovaries, plays a crucial role in regulating the menstrual cycle and breast development in individuals. Furthermore, it is important to note that although breast cancer is less common in males, men can also be affected by the disease. It works with estrogen, preparing the breast tissue for potential pregnancy and supporting the growth and differentiation of mammary gland cells [25].

Mechanisms of hormonal imbalance and its impact on breast tissue

Hormonal imbalance is an abnormality or disruption in the body’s optimal levels or ratios of hormones. However, it is important to note that defining a specific numerical ratio or range of hormones as “balanced” or “normal” can be challenging due to individual variations and the complexity of hormonal interactions [25].

One mechanism by which hormonal imbalance contributes to breast cancer is activating estrogen receptors (ERs) and progesterone receptors (PRs) in breast cells. Upon binding of estrogen to ER or progesterone to PR, a complex series of intracellular signaling events is initiated, which can contribute to cell proliferation regulation and cell death inhibition. In excess estrogen or impaired progesterone signaling, these pathways can become dysregulated, resulting in uncontrolled cell growth and the formation of tumors [26,27].

Furthermore, hormonal imbalances can also influence the microenvironment of the breast tissue [28]. Estrogen, for example, promotes the growth of blood vessels and stimulates the production of growth factors that facilitate tumor angiogenesis and metastasis. It can also impact the immune response within the breast, potentially modulating tumor immune surveillance and promoting tumor evasion of the immune system [28,29].

Role of hormone receptors and their significance in breast cancer

Hormone receptors, such as ERs and PRs, play a critical role in breast cancer development. The presence or absence of these receptors in breast cancer cells helps classify tumors into different subtypes and guides treatment decisions [30,31].

ER-positive (ER+) breast cancer refers to tumors that express ERs on their cell surface. These tumors are dependent on estrogen signaling for growth and proliferation. ER+ breast cancer is the most common subtype of breast cancer and often responds well to hormonal therapies that target estrogen signalings, such as selective ER modulators (SERMs) or aromatase inhibitors (AIs) [32,33]. PR-positive (PR+) breast cancer indicates the presence of PRs on the tumor cells. The expression of PRs may provide additional information about tumor behavior and response to treatment [34].

The presence of hormone receptors helps classify breast cancer subtypes and has prognostic implications. Hormone receptor status can influence the aggressiveness of the tumor, response to treatment, and overall prognosis. Hormone receptor-negative breast cancer, which lacks estrogen and PRs, tends to be more aggressive and less responsive to hormonal therapies [35,36]. Understanding the role of hormone receptors in breast cancer is essential for personalized treatment approaches, as targeting these receptors with specific therapies can effectively inhibit tumor growth and improve patient outcomes [37].

Estrogen and breast cancer

Estrogen, an essential female sex hormone, plays a significant role in breast cancer development. It promotes the growth and development of breast tissue and regulates various cellular processes, including cell proliferation, differentiation, and survival. However, excessive or prolonged exposure to estrogen can disrupt the delicate balance of cell growth and death, leading to the initiation and progression of breast cancer [38]. Estrogen can be derived from both endogenous and exogenous sources. Endogenous estrogen is primarily produced in the ovaries, while exogenous estrogen can come from HRT or environmental sources, such as certain plastics and pesticides [39].

Estrogen metabolites and their impacts on breast tissue

In the realm of estrogen metabolism, the body engages in processes that involve the breakdown and elimination of estrogen. Two primary pathways, hydroxylation and methylation, play a pivotal role in estrogen metabolism. These pathways give rise to distinct estrogen metabolites, with certain metabolites being linked to diverse effects on breast tissue [40,41]. One important metabolite is 16α-hydroxy estrone (16α-OHE1), considered a more potent estrogen than the parent hormone estradiol. High levels of 16α-OHE1 have been linked to an increased risk of breast cancer, as this metabolite has been shown to promote cell proliferation and DNA damage [42,39].

On the other hand, 2-OHE1 is a weaker estrogen and is believed to have anti-proliferative and anti-carcinogenic properties. This metabolite is associated with a lower risk of breast cancer. The balance between the production of 16α-OHE1 and 2-OHE1 is crucial in maintaining the normal physiological effects of estrogen in breast tissue [43]. The interplay between estrogen metabolism and breast cancer risk is complex and is influenced by various factors, including genetics, lifestyle choices, and environmental exposures. Understanding the impact of estrogen metabolism on breast tissue can provide insights into the underlying mechanisms of estrogen-related breast cancer development [44].

ER+ breast cancer and hormone therapies

ER+ breast cancer refers to tumors that express ERs on their cell surface. These receptors allow estrogen to bind and activate signaling pathways that promote cell growth and survival. ER+ breast cancer accounts for a significant proportion of breast cancer cases [45]. Hormone therapies that target estrogen signaling pathways have been developed to treat ER+ breast cancer. SERMs, such as tamoxifen and raloxifene, are commonly used in adjuvant and preventive settings. These drugs block estrogen binding to the receptors, inhibiting estrogen-mediated cell growth and reducing the risk of recurrence [45].

AIs, such as anastrozole, letrozole, and exemestane, are another class of hormone therapy used in postmenopausal women with ER+ breast cancer. AIs reduce estrogen production by blocking the enzyme aromatase, which converts androgens into estrogens. By suppressing estrogen production, AIs help inhibit the growth of ER+ breast cancer cells [46]. Hormone therapies have proven effective in reducing the risk of recurrence and improving survival rates in ER+ breast cancer patients. To provide more insightful information, it would be beneficial to include specific details regarding the efficacy and side effects experienced by individuals. It is important to note that these treatments’ effectiveness and potential adverse reactions can differ significantly among individuals. This emphasizes the significance of adopting personalized treatment strategies that consider factors such as hormone receptor status, patient characteristics, and potential biomarkers [47].

Progesterone and breast cancer

Role of Progesterone in Breast Cancer Development

Progesterone, another key hormone in the female reproductive system, plays a significant role in breast cancer development. It acts in conjunction with estrogen to regulate the growth and differentiation of breast tissue. Progesterone promotes the proliferation of mammary epithelial cells and supports the development of the mammary gland in preparation for a potential pregnancy [48].

However, like estrogen, an imbalance in progesterone levels or disrupted progesterone signaling can contribute to the development of breast cancer. Excessive exposure to progesterone, particularly in combination with estrogen, can lead to uncontrolled cell growth and the formation of tumors. The precise mechanisms through which progesterone influences breast cancer development are currently under active investigation. However, it is well-established that progesterone plays a crucial role within the intricate hormonal network involved in the pathogenesis of breast cancer [49].

PR+ Breast Cancer and Its Implications

PR+ breast cancer refers to tumors that express PRs on their cell surface. The presence of PRs indicates that these tumors can be influenced by progesterone signaling. PR+ breast cancer accounts for a substantial proportion of breast cancer cases, often co-existing with ER+ tumors [50].

The presence of PR in breast cancer cells has both prognostic and predictive implications. PR expression has been associated with a more favorable prognosis and increased responsiveness to hormonal therapies. Patients with PR+ tumors may have better outcomes and higher rates of response to hormone-based treatments, such as anti-estrogen therapy or chemotherapy combined with hormonal therapy [51]. The assessment of PR status and ER status help further categorize breast cancer subtypes and guide treatment decisions, enabling personalized therapeutic approaches for patients.

Interaction Between Estrogen and Progesterone in Breast Cancer Progression

Estrogen and progesterone interact closely in the development and progression of breast cancer. ERs and PRs in breast cancer cells allow for complex cross-talk between these hormonal pathways [52]. Estrogen, through ER signaling, stimulates the expression of PR in breast tissue. The binding of estrogen to ER can lead to increased PR expression, resulting in enhanced sensitivity to progesterone and its proliferative effects [53].

Conversely, progesterone can influence estrogen signaling by modulating the expression and activity of ER. Progesterone can induce the expression of co-regulatory proteins that interact with ER, affecting its transcriptional activity and subsequent effects on cell growth and survival [54]. The interplay between estrogen and progesterone signaling pathways can influence breast cancer progression and therapeutic responses. Understanding the complex interactions between these hormones is essential for developing targeted treatment strategies that address both estrogen and progesterone signaling pathways in breast cancer [55].

Other hormones and breast cancer

While estrogen and progesterone are key hormones in breast cancer development, other hormones and growth factors also play important roles in breast cancer pathogenesis. These include insulin, insulin-like growth factors (IGFs), and growth hormone (GH) [56]. Insulin, a hormone produced by the pancreas, regulates blood sugar levels. However, insulin also has mitogenic effects and can promote cell growth and proliferation. Elevated insulin levels or resistance, as seen in conditions like obesity and type 2 diabetes, have been associated with an increased risk of breast cancer [57]. Insulin can interact with IGF-1, activating signaling pathways that promote cell survival and tumor growth [57].

IGFs, particularly IGF-1, are growth factors that share structural similarities with insulin. They play a crucial role in normal growth and development. However, excessive IGF-1 levels or altered IGF signaling can contribute to breast cancer development. IGF-1 can stimulate cell proliferation, inhibit apoptosis (programmed cell death), and promote angiogenesis (formation of new blood vessels) in breast tissue [58]. GH, produced by the pituitary gland, regulates growth and metabolism. It interacts with IGF-1 and influences breast tissue development. GH secretion or responsiveness alterations can impact breast cancer risk [56,59].

Cross-talk between different hormone pathways and their contribution to breast cancer

Hormone pathways in breast cancer are interconnected, and cross-talk between these pathways can influence tumor development and progression. For example, estrogen can stimulate the production of insulin and IGFs, creating a positive feedback loop that promotes cell growth and survival in breast tissue. Estrogen can also modulate the expression and activity of GH receptors [60]. Estrogen and progesterone signaling pathways can also interact with other hormone receptors, such as the androgen receptor (AR). Androgens, including testosterone, can be converted into estrogen in breast tissue and contribute to estrogen signaling. The involvement of androgens in male breast cancer, if any, remains a topic of interest. Existing studies have demonstrated the participation of AR in both ER+ and ER-negative (ER-) breast cancers. Activation of the AR has been shown to impact tumor growth and the response to therapy [61,62].

Furthermore, the interplay between hormonal pathways and other molecular signaling networks, such as the human epidermal growth factor receptor 2 (HER2) pathway, can also impact breast cancer development. HER2-positive breast cancers, characterized by the overexpression of the HER2 receptor, can exhibit cross-talk with hormone receptors and influence the response to hormonal therapies [63]. Understanding the complex interactions and cross-talk between different hormone pathways is vital for comprehending the multifaceted nature of breast cancer and developing targeted therapies that simultaneously address multiple hormonal signaling networks [64].

Hormonal imbalance and breast cancer risk assessment

Hormonal Markers for Breast Cancer Risk Assessment

Hormonal markers, such as hormone receptor status, are crucial in assessing breast cancer risk. Determining the presence or absence of hormone receptors on breast cancer cells helps classify tumors into different subtypes and provides important information for prognosis and treatment decisions [65]. ER status is a commonly evaluated hormonal marker in breast cancer. ER+ tumors express ERs on their cell surface, indicating that they depend on estrogen signaling for growth and proliferation. ER- tumors, on the other hand, do not express ERs and may have different underlying molecular characteristics [66].

PR status is another hormonal marker evaluated in breast cancer. PR+ tumors express PRs, suggesting their potential responsiveness to progesterone signaling. PR-negative (PR-) tumors lack PRs and may exhibit distinct molecular features [67]. Determining hormone receptor status and other biomarkers like HER2 status helps classify breast cancer subtypes and guides treatment decisions. Hormone receptor-positive (ER+/PR+) tumors are often candidates for hormonal therapies, such as SERMs or AIs. In contrast, hormone receptor-negative (ER-/PR-) tumors may require alternative treatment approaches [68].

Importance of Hormone Testing in Breast Cancer Diagnosis and Treatment Decisions

Hormone testing, including the assessment of hormone receptor status, is of utmost importance in breast cancer diagnosis and treatment decisions. It provides valuable information for personalized treatment approaches and prognostic evaluation [69]. In the diagnostic phase, hormone receptor testing helps confirm the presence of hormone receptor-positive (HR+) or negative breast cancer. This information aids in distinguishing different subtypes of breast cancer, which have varying biological characteristics and clinical behaviors. It assists in determining the appropriate treatment strategies and predicting the likelihood of response to specific therapies [70].

Hormone receptor status also influences treatment decisions in the adjuvant and neoadjuvant settings. Patients with HR+ tumors may benefit from hormonal therapies alone or in combination with other treatments. These therapies aim to block or interfere with hormone signaling pathways, suppressing tumor growth and reducing the risk of recurrence [71]. Moreover, hormone receptor status serves as a prognostic factor, providing information about the potential aggressiveness of the tumor and the likelihood of disease progression. Patients with HR+ tumors tend to have better outcomes than those with hormone receptor-negative tumors, as they are more likely to respond to hormonal therapies [72].

Hormonal therapies for breast cancer

Hormonal therapies are crucial in treating HR+ breast cancer, particularly ER+ tumors. These therapies aim to interfere with hormonal signaling pathways and block the effects of estrogen or progesterone on tumor growth and progression [73].

SERMs, such as tamoxifen and raloxifene, are commonly used hormonal therapies. They act as competitive inhibitors of estrogen binding to the ER and exert both agonistic and antagonistic effects depending on the tissue. In the breast, SERMs block estrogen-mediated cell growth and reduce the risk of recurrence. Tamoxifen is widely used in pre- and postmenopausal women, while raloxifene is primarily used for breast cancer prevention [74].

AIs, including anastrozole, letrozole, and exemestane, are another class of hormonal therapies. AIs block the enzyme aromatase, which converts androgens into estrogens. By reducing estrogen production, AIs effectively inhibit estrogen-mediated cell growth. AIs are primarily used in postmenopausal women with HR+ breast cancer [75].

Other hormonal therapies include selective ER downregulators (SERDs) like fulvestrant, which binds to the ER and promotes its degradation, and luteinizing hormone-releasing hormone (LHRH) agonists, such as goserelin, which suppress ovarian function and reduce estrogen production [76].

Benefits and limitations of hormonal therapies

Hormonal therapies have several benefits in treating HR+ breast cancer [77]. They have effectively reduced the risk of recurrence, improved overall survival, and provided long-term benefits. Hormonal therapies are generally well-tolerated, with fewer severe side effects than chemotherapy. They can be administered orally or by injection, allowing for convenient outpatient treatment [77].

However, hormonal therapies also have limitations. Some patients may experience side effects such as hot flashes, vaginal dryness, and mood changes. SERMs, particularly tamoxifen, can increase the risk of endometrial cancer and blood clots. AIs may lead to bone loss and joint pain in some individuals. Additionally, not all patients with HR+ breast cancer respond equally to hormonal therapies, and resistance may develop over time, necessitating alternative treatment approaches [78].

Future directions in hormonal therapy research

In recent years, research in hormonal therapies for breast cancer has focused on optimizing treatment strategies and overcoming resistance. Ongoing studies investigate the efficacy of extended hormonal therapy durations beyond five to 10 years, explore combination therapies with targeted agents and immunotherapies, and identify potential biomarkers to predict treatment response.

Emerging research areas include the development of novel hormonal therapies with improved efficacy and tolerability profiles. For instance, selective ER degraders (SERDs) are being investigated as a new hormonal therapy class that completely blocks estrogen signaling by degrading the ER. Additionally, novel drugs targeting other components of the hormonal signaling pathways, such as cyclin-dependent kinase (CDK) inhibitors, are promising in combination with hormonal therapies. Personalized medicine approaches are also being explored to identify predictive biomarkers to help guide treatment decisions and select patients most likely to benefit from specific hormonal therapies.

## Conclusions

In conclusion, this comprehensive review has shed light on the significant role of hormonal imbalance in the development of breast cancer. The findings have highlighted the importance of understanding the complex interplay between hormones, hormone receptors, and other molecular signaling pathways in breast tissue. Estrogen and progesterone, in particular, have been identified as major players in breast cancer development, with ER+ and PR+ tumors requiring targeted hormonal therapies. The review has also emphasized the involvement of other hormones, such as insulin and growth factors, and their cross-talk with hormone pathways in breast cancer progression. Further research is needed to unravel the intricate mechanisms underlying hormonal imbalances and their contribution to breast cancer. This includes exploring novel hormonal therapies, personalized medicine approaches, and integration of hormonal imbalance assessment into risk assessment models. By advancing our understanding and optimizing therapeutic strategies, we aim to improve outcomes for individuals affected by breast cancer and pave the way for future innovative prevention and treatment interventions.
